# Non-Thermal Plasma Treatment of Poly(tetrafluoroethylene) Dental Membranes and Its Effects on Cellular Adhesion

**DOI:** 10.3390/ma16206633

**Published:** 2023-10-10

**Authors:** Vasudev Vivekanand Nayak, Nicholas Alexander Mirsky, Blaire V. Slavin, Lukasz Witek, Paulo G. Coelho, Nick Tovar

**Affiliations:** 1 Department of Biochemistry and Molecular Biology, Miller School of Medicine, University of Miami, Miami, FL 33136, USA; vxn188@miami.edu (V.V.N.); nmirsky@med.miami.edu (N.A.M.); bvs11@miami.edu (B.V.S.); pgc51@med.miami.edu (P.G.C.); 2 Biomaterials Division, College of Dentistry, New York University, New York, NY 10010, USA; nicktovar@gmail.com; 3 Department of Biomedical Engineering, Tandon School of Engineering, New York University, 6 MetroTech Center, Brooklyn, NY 11201, USA; 4 Hansjörg Wyss Department of Plastic Surgery, Grossman School of Medicine, New York University, New York, NY 10017, USA; 5 DeWitt Daughtry Family Department of Surgery, Division of Plastic Surgery, Miller School of Medicine, University of Miami, Miami, FL 33136, USA; 6 Department of Oral and Maxillofacial Surgery, New York University, Langone Medical Center and Bellevue Hospital Center, New York, NY 10016, USA

**Keywords:** guided bone regeneration, non-thermal plasma, dental membranes

## Abstract

Non-resorbable dental barrier membranes entail the risk of dehiscence due to their smooth and functionally inert surfaces. Non-thermal plasma (NTP) treatment has been shown to increase the hydrophilicity of a biomaterials and could thereby enhance cellular adhesion. This study aimed to elucidate the role of allyl alcohol NTP treatment of poly(tetrafluoroethylene) in its cellular adhesion. The materials (non-treated PTFE membranes (NTMem) and NTP-treated PTFE membranes (PTMem)) were subjected to characterization using scanning electron microscopy (SEM), contact angle measurements, X-ray photoelectron spectroscopy (XPS), and electron spectroscopy for chemical analysis (ESCA). Cells were seeded upon the different membranes, and cellular adhesion was analyzed qualitatively and quantitatively using fluorescence labeling and a hemocytometer, respectively. PTMem exhibited higher surface energies and the incorporation of reactive functional groups. NTP altered the surface topography and chemistry of PTFE membranes, as seen through SEM, XPS and ESCA, with partial defluorination and polymer chain breakage. Fluorescence labeling indicated significantly higher cell populations on PTMem relative to its untreated counterparts (NTMem). The results of this study support the potential applicability of allyl alcohol NTP treatment for polymeric biomaterials such as PTFE—to increase cellular adhesion for use as dental barrier membranes.

## 1. Introduction

In the field of oral and maxillofacial reconstruction, bone deficiency and resulting bone atrophy remains a major concern. Such irregularities originate from a variety of conditions including systemic and periodontal diseases, tooth loss, trauma, and pathology, among others [1,2,3,4,5]. To improve bone defect rehabilitation, and to provide an anatomically pleasing ridge contour, guided bone regeneration (GBR) procedures have been mostly utilized as a treatment modality [1,6,7,8,9]. Owing to its successful surgical outcomes, this modality has become ubiquitous in the repair of alveolar ridge defects over the last seven decades [10,11,12,13]. GBR relies on the barrier membrane-based isolation of bone defect volume, thereby allowing osteoprogenitor cells to repopulate defect sites by simultaneously preventing the entry of rapidly proliferating non-osteogenic (soft tissue) cells within the oral cavity [1,6,7,8].

This core principle of GBR membranes, namely, to serve as a protective barrier, also helps to ensure site stability [14]. Intrinsically, resorbable barrier membranes, like those made from collagen, can be broken down via hydrolysis and absorbed by the body rapidly, thereby potentially compromising the healing process [15,16]. On the other hand, non-resorbable barrier membranes, such as those fabricated using poly(tetrafluoroethylene) (PTFE), have reproducible physicochemical properties, and allow for favorable surgical outcomes by ensuring predictable, compartmentalized healing [17,18]. As such, a key component of the long-term success of GBR stems from the membrane’s ability to integrate with native cells at the host site. Unfortunately, a major concern associated with the use of PTFE in the oral cavity is its poor bonding capacity to surrounding tissue structures due to low adhesion properties, as PTFE membranes have been shown to dehisce in up to ~70% of cases [19,20,21]. Its smooth, hydrophobic surface is not conducive to cellular adhesion of native progenitor cells and necessitates the modification of surface characteristics [19].

While researchers have thoroughly studied osteointegration at the bone–membrane interface, there has been a notable a shift in interest toward the external epithelial barrier [22]. A strong peri-implant barrier has been shown to be facilitated through cellular adhesion, replicating the host’s native architecture [23,24,25]. Adhesion improves membrane stability, particularly in unfavorable areas such as the oral cavity, where horizontal, vertical, and torsional forces are prevalent. In contrast, the absence of membrane stability results in potential socket exposure to bacteria and/or epithelial tissue infiltration, as described above, which can consecutively disrupt the mechanical and histological integrity of regenerated tissue [26]. Recent studies have modified membrane surfaces by means of acid etching, growth factor coating, or non-thermal plasma (NTP) treatment [27]. Each treatment affects the surface energy (SE), chemical properties, and/or surface topography of the membrane differently, thereby altering the amount of cell attachment and cell growth uniformity on the membrane surface. Improved cellular adhesion through membrane modification ultimately allows for more effective tissue regeneration [23,28,29,30,31,32].

NTP treatment of biomaterials has the potential to increase the hydrophilicity (or SE) of the membrane surface via physical and chemical mechanisms, which in turn increases the adhesion of proteins necessary for the attachment and proliferation of cells. The effects of plasma treatment of metals and ceramics on cell proliferation and adhesion have been previously investigated. For example, titanium dental implants treated with plasma exhibited increased SE and greater osteointegration [33,34,35,36]. While NTP alters SE, another advantage is its capacity to modify polymeric materials via unique physical and chemical interventions. In addition, unlike other surface treatments, plasma gas can be incorporated with a variety of organic and inorganic compounds like allyl alcohol—a monomer that can be polymerized upon deposition [37]. The deposition of a thin monomer layer has been shown to improve the surface functionality of the underlying substrates [37,38,39]. However, scientific literature on cellular adhesion as a result of NTP treatment of PTFE dental membranes is scarce [40,41,42,43,44].

The objective of this proof-of-concept study was to investigate alterations in cell adhesion to PTFE membranes subjected to NTP treatment with an allyl alcohol monomer. To test this phenomenon, we assessed the adhesive and proliferative characteristics of three distinct surface specimens: PTFE membranes treated with plasma (PTMem), untreated PTFE membranes (NTMem), and glass coverslips (glass—negative control), seeded with cells. In summary, the results of this study signify that a surface conducive to cellular attachment was achieved via the NTP process in a safe and effective manner. Furthermore, the proposed technique could serve as a steppingstone for the application of the NTP process to other commonly utilized polymeric dental membrane surfaces.

## 2. Materials and Methods

### 2.1. NTP Treatment

Three types of surface samples were used in this study: (i) 22 × 22 mm glass coverslips (glass—negative control) (FisherScientific, Hampton, NH, USA); (ii) untreated, non-resorbable, high-density PTFE membranes (Cytoplast TXT-200, 12 × 24 mm, Osteogenics Biomedical, Inc., Lubbock, TX, USA) (abbreviated: NTMem); and (iii) NTP treated, non-resorbable, high-density PTFE membranes (Cytoplast TXT-200, 12 × 24 mm, Osteogenics Biomedical, Inc., Lubbock, TX, USA) (abbreviated: PTMem) [45]. The PTFE membranes (NTMem and PTMem) were sectioned in half to make 12 × 12 mm sections, which were then placed into well plates for further processing. Allyl alcohol monomer NTP treatment of the PTFE membranes was performed using a previously developed protocol [38] and a descriptive application technique developed and patented by Osteogenics Biomedical, Inc. (Lubbock, TX, USA) [46]. In brief, plasma was emitted by a microwave plasma generator of 2.45 GHz frequency in a quartz tube at the top of a reactive chamber. Membranes were attached to the table in the post-discharge area and pulsed plasma with a pulse frequency of 125 Hz and 25% of duty time was applied. Plasma parameters were set at a power of 60–180 W and an initial pressure of 5 × 10^−3^ mbar (monomer pressure: 1 mbar).

### 2.2. Surface Characterization

#### 2.2.1. Scanning Electron Microscopy (SEM) and Contact Angle Measurements

The two polymeric experimental groups, NTMem and PTMem, were subjected to chemical characterization and an in vitro analysis. Initially, the two groups of PTFE membranes were assessed via SEM (Hitachi S-3500N, Hitachi, Tokyo, Japan) at 5 kV in secondary electron mode to ascertain differences in surface topography as a result of the etching treatment. To assess the SE of both membranes (*n* = 6/group), the Owens–Wendt–Rabel–Kaelble (OWRK) method was utilized [47]. We deposited 500 μL droplets of distilled water, ethylene glycol, and diiodomethane individually onto the surface of each implant group with a micro-pipette (OCA30, Data Physics Instruments GmbH, Filderstadt, Germany). Images were captured and analyzed using software (SCA30, v3.4.6 build 79, Data Physics Instruments GmbH, Filderstadt, Germany). The relationship between the contact angle and SE was determined, and the SE was calculated using the following equation:

(1) $$\gamma_{L}=\gamma_{L}^{D}+{\;\gamma }_{L}^{P}$$

where γ_L_ is the SE, $\gamma_{L}^{D}$ is the dispersive component, and $\gamma_{L}^{P}$ is the polar component [48].

#### 2.2.2. X-ray Photoelectron Spectroscopy (XPS)/Electron Spectroscopy for Chemical Analysis (ESCA)

Surface-specific chemical assessment was performed via XPS and ESCA. The NTMem and PTMem (*n* = 3/group) were inserted into a vacuum transfer chamber and degassed to 10^−7^ torr. The samples were then transferred under vacuum conditions to a PHI 5701LSci instrument (Physical Electronics, Inc., Chanhassen, MN, USA). Survey and high-resolution spectra were obtained using a 165 mm mean radius concentric hemispherical analyzer. The analyzer was operated at a constant pass energy of ~1500 eV for surveying and ~285 eV for high-resolution scanning. The acceptance angle was set to ±7° and the take-off angle was set to 50°, with a spot size of 2000 μm × 800 μm. The membrane surfaces were evaluated at various locations to measure for atomic concentrations of different elements as well as carbon chemical state assignments.

### 2.3. In Vitro Characterization

#### 2.3.1. Cell Passaging

Human Embryonic Kidney 293 cells (HEK) exhibit epithelial morphology, are adherent, and have been previously utilized in the literature pertaining to the in vitro characterization of dental membranes for bone and tissue regeneration [49]. Cells were obtained from Sigma Aldrich (St. Louis, MO) and were allowed to reach 70–80% cell confluency in culture medium (comprising Dulbecco’s modified eagle medium with high glucose, 10% fetal bovine serum (FBS), 1% penicillin streptomycin, and 1% glutamine) prior to subjecting to passaging. For passaging, the culture medium was removed, and cells were washed with 8 mL of PBS and then aspirated. Subsequently, 1 mL of trypsin was added to the flask to promote cell dissociation from the surface [50]. Fresh culture medium (15 mL per new flask) was added to neutralize the trypsin and to wash the cell culture surface. The solution was distributed evenly to new flasks to promote monolayer cell growth.

#### 2.3.2. Fluorescent Cell Membrane Labeling

Prior to seeding cells on the membranes, the cells were fluoresced according to the manufacturer’s guidelines for the PKH26 Red Fluorescent Cell Linker Kit (Sigma Aldrich, St. Louis, MO, USA) for general cell membrane labeling. Cell work was performed in the dark to minimize the loss of fluorescence intensity. To confirm the concentration of cells, cell counting was performed using trypan blue and a hemocytometer. The required volume of cell solution containing 10^7^ cells was calculated, and the suspension was placed in a conical tube and washed once using culture medium without FBS. The solution was centrifuged at 400× *g* for 5 min to create a loose pellet, and the supernatant was carefully aspirated. The pellet was resuspended in 0.5 mL of diluent C to create a 2× cell suspension. A 2× dye solution was prepared by combining 2 μL of the PKH26 ethanolic dye solution and 0.5 mL of diluent C. Immediately, 0.5 mL of 2× cell suspension was added to 0.5 mL of 2× dye solution, rapidly mixed, and then incubated for 5 min. To stop the staining process, 5 mL of complete medium was added. The cells were centrifuged (at 400× *g*) for 10 min at room temperature, after which the supernatant was removed. The cell pellet was resuspended in 10 mL of culture medium and centrifuged once again at 400× *g* for 5 min. The pellet was washed twice with 10 mL of culture medium to remove unbound dye. After the final wash, the cell pellet was resuspended in 15 mL of culture medium, transferred to a cell culture flask, and placed in the incubator for subsequent experimentation.

#### 2.3.3. Adhesion and Proliferation Experiments

Each trial had three time points (1, 4, and 8 days), with each time point consisting of three 6-well plates, each containing one of three types of surface samples: glass coverslips, NTMem, or PTMem. With 54 wells per trial, a total of 108 wells (two trials) were used in the study. At least 24 h prior to seeding, the glass, NTMem, and PTMem samples were soaked in complete medium in the wells. On day 0, the cells were transferred from the flask(s) into a 15 mL tube, and cell density was determined using a hemocytometer. The seeding volume was calculated to include 10^5^ cells. To ensure the homogeneity of the cell solution during seeding, the solution was intermittently mixed using a shaker. At the 1-day time point, a surface sample from one well of each plate was used for obtaining pictographs. Each surface sample was inverted and placed in a 60 × 15 mm petri dish with Hanks Balanced Salt Solution (HBSS) to preserve cell viability for imaging. The remaining five wells of each surface sample were prepared for cell counting. Briefly, using forceps, each glass coverslip was removed from its well and submerged in a beaker of PBS, and treated with 100 μL of trypsin for 3 min, following which complete medium was added to the wells and mixed until homogenous. The total number of cells per mm^2^ was calculated for each glass sample after performing counting with a hemocytometer. The procedure was repeated for both the NTMem and PTMem samples; and at the 4- and 8-day time points.

## 3. Statistical Analysis

Mean cell counts (log_10_/mm^2^) obtained at each time point in vitro from the different experimental groups were compared through *t*-tests. SE measurements were compared using a non-parametric independent samples Mann–Whitney U Test. Statistical analyses were performed on IBM SPSS (v29, IBM Corp., Armonk, NY, USA), and *p* < 0.05 was considered statistically significant.

## 4. Results

**Figure 1:**
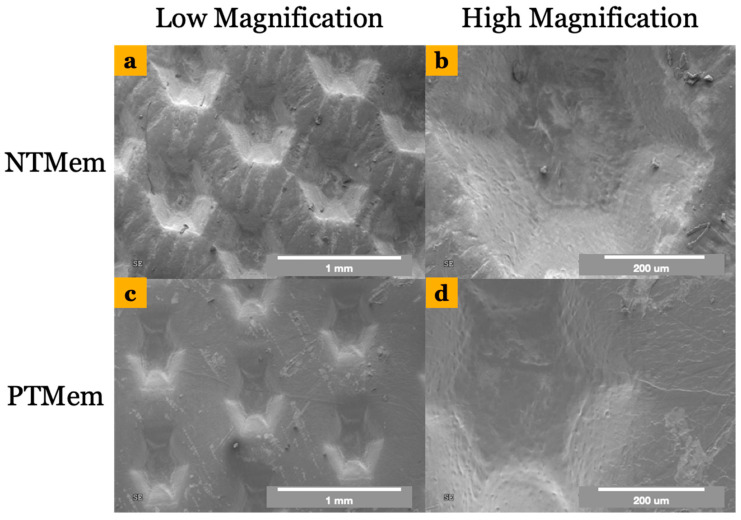
Scanning electron micrographs showing the topography of (**a**,**b**) the NTMem surface at low (~50×) and high (~200×) magnifications, respectively; and (**c**,**d**) the PTMem surface at low (~50×) and high (~200×) magnifications, respectively.

**Figure 2:**
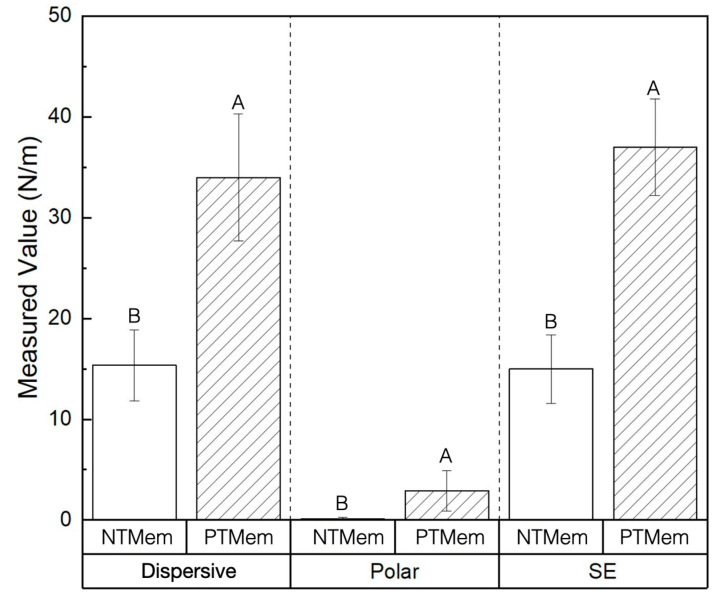
SE measurements of the NTMem and PTMem groups and their respective dispersive and polar components obtained using the OWRK method. Data in the plots are presented as means along with respective 95% confidence interval values (mean ± 95% CI), with *p* < 0.05 considered statistically significant. The letters indicate statistically homogenous groups.

Samples for NTMem and PTMem comprised a smooth surface side and a texturized surface side, with the latter intended to be in direct contact with soft tissue. Qualitative characterization was hence performed on the textured surface of the samples. SEM micrographs showed a difference in surface morphology between both membranes, where NTMem samples (Figure 1a,b) appeared rougher relative to the smoother morphology of PTMem samples (Figure 1c,d). SE, dispersive, and polar components were measured for each of the two experimental groups (*n* = 6/group) and are presented in Table 1 and depicted in Figure 2. The OWRK method revealed a significantly higher SE in PTMem samples (37 ± 4.8 N/m) relative to NTMem samples (15 ± 3.4 N/m) (*p* = 0.02).

XPS detailed atomic concentrations (Table 2) and carbon chemical states (Table 3) for each measured analysis area of the NTMem and PTMem samples (n=3/group). Atomic concentration data showed peaks of carbon (C), oxygen (O), fluorine (F), silicon (Si), and chlorine (Cl) for both membranes tested. The NTMem samples predominantly contained C and F, with low levels of O and Si, and possibly Cl contamination. The PTMem samples added O to the structure and reduced F concentration. The PTMem samples also contained low levels of Si and possibly Cl contamination. Non-linear least-squares (NLLS) curve fitting was applied to carbon high-resolution spectra to assist in chemical state assignment. The carbon spectra were curve-fitted using seven component peaks: C-C, C-O, C=O, O-C=O, CF, CF_2_, and CF_3_. Carbon spectra data showed defluorination and O incorporation after NTP treatment of the membrane samples (detailed in Table 3).

**Figure 3:**
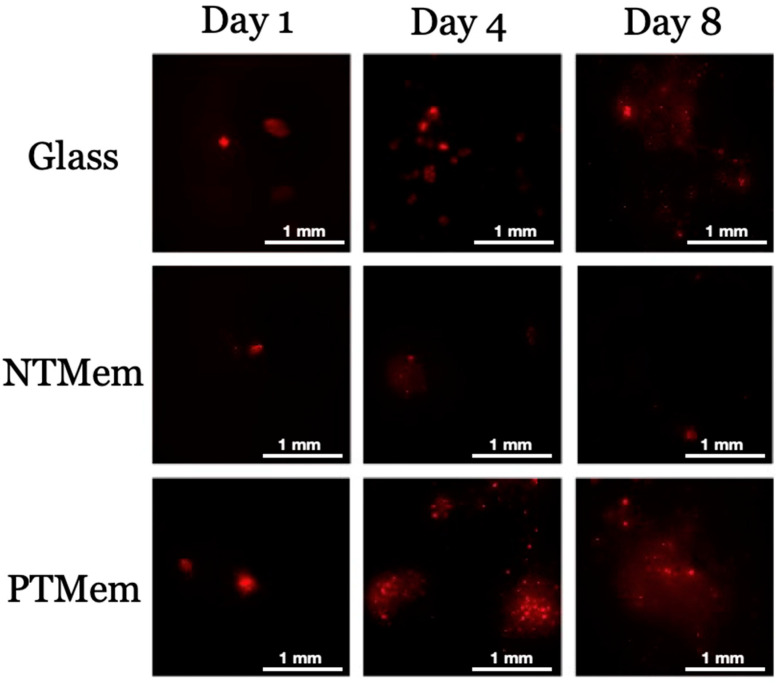
Representative pictographs of the fluorescence labeling (PKH26 Red) performed upon cells seeded on the different experimental surfaces used in this study at the three time points (1, 4, and 8 days).

**Figure 4:**
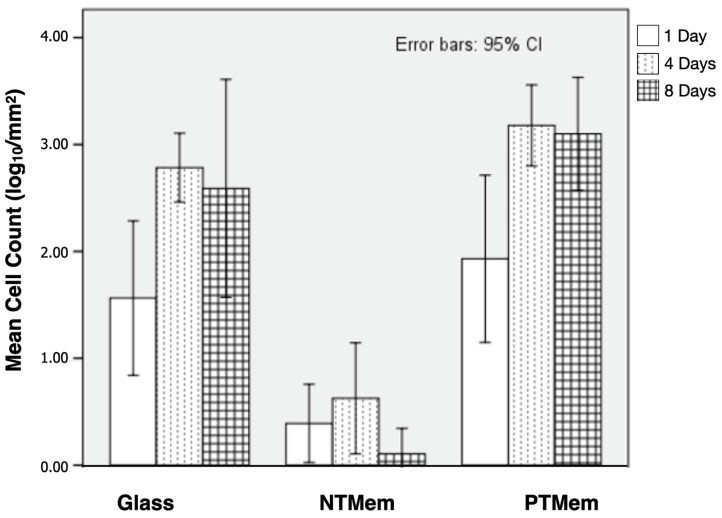
Mean cell counts (log_10_/mm^2^) upon the various experimental surfaces, evaluated at different time points. Data in the plots are presented as means along with respective 95% confidence interval values (mean ± 95% CI).

Qualitatively, the raw imaging data (Figure 3) collected from the fluorescence analysis of the different surfaces and their corresponding times in vitro indicated greater fluorescence (the presence cells) on the PTMem samples relative to NTMem samples at all time points evaluated. Additionally, the fluorescence of PTMem samples was analogous to that of glass (negative control) at day 1, with the former being higher at days 4 and 8. Quantitatively, Figure 4 shows the statistical summary of the log_10_-transformed data of cell counts over the samples’ surface as a function of time. Consistent with the sample fluorescence images, the mean cell counts suggested relatively lower adhesion of cells to the NTMem surfaces, <10 cells/mm^2^. By contrast, the glass and PTMem surfaces both showed at least 10–100 cells/mm^2^ at earlier incubation times, and ~1000 cells/mm^2^ at longer incubation times. From statistical analysis, the mean cell count (log_10_/mm^2^) for the NTMem samples at all incubation times lay outside the confidence bounds of the other materials, indicating significantly less adhesion to this surface (*p* < 0.05). It is important to note that no differences in cell counts (log_10_/mm^2^) were observed between PTMem and glass surfaces at any of the time points tested (*p* > 0.05).

## 5. Discussion

The successful healing of defects in GBR is dependent on a coordinated series of events that are strongly dependent on the functionality of the implanted membrane [1]. An ideal dental barrier membrane is conducive to cellular attachment; will not induce an immunologic response; and is convenient to use [9]. There are well documented studies of the effects of modification of barrier membranes for osteointegration and cellular adhesion, but studies highlighting the benefits of membrane modification at the epithelial junction are limited [7,9,51]. Modification of polymer surfaces that constitute the barrier membrane can be achieved via several techniques. These include but are not limited to acid/alkali treatments and radiation treatments (such as ultraviolet radiation) [52]. However, recent efforts in the NTP treatment of polymer surfaces have shown promising outcomes for surface modification as the process is solvent-free and does not alter the material’s bulk characteristics [52,53,54].

The current study aimed to investigate the effect of allyl alcohol NTP treatment on the in vitro performance (cellular adhesion and proliferation) of membranes synthesized using PTFE—a hydrophobic surface [21]. In order to achieve a hydrophilic surface capable of ensuring cellular adhesion and proliferation, the precise control of surface properties is of paramount importance. Contact angle ($\theta_{c})$, a direct measure of hydrophilicity or hydrophobicity, is a surface property previously shown to be tailorable using NTP treatment [55,56]. Small contact angles correspond to highly wettable surface (hydrophilicity), while the opposite is true for a hydrophobic surface [55]. Mathematically, the widely used OWRK method indicates an inversely proportional relationship between ($\theta_{c})$ and SE [47]. In the context of the current study, the induced NTP treatment therefore resulted in a significant increase in SE, in turn improving the hydrophilicity of the underlying substrate (PTFE).

To elaborate, using the OWRK approach to quantify SE can be expressed as a sum of dispersive and polar components. The dispersive component of SE characterizes the nonpolar interactions between the surface and the dispensed liquid and is determined by factors such as surface topography [57]. On the other hand, the polar component of SE characterizes the polar interaction between the surface of the material and the working fluid and is determined by the presence of polar groups, electric charges, and free radicals on the surface [34]. In this study, qualitative observation of the membranes via SEM showed that NTMem samples presented a rougher surface texture relative to PTMem samples, possibly due to erosion of the surface and subsequent redeposition of etching fragments during NTP treatment, thereby increasing the dispersive component of SE [58]. The redeposition of the etching fragments also increased the polar component of the PTMem samples due to the incorporation of reactive functional groups present on the surface [59].

Specifically, the increase in the polar component during NTP treatment resulted in a change in surface chemistry as partial defluorination of PTFE by CF and CF_x_ polymer chain breakage was observed by XPS and ESCA. This process could have allowed for the ion interaction of radicals on the polymer surface, such as air oxygen, alcohol, and -C=C- bonds [60]. Of note is the 45-fold increase in C-C and the 58-fold increase in C-O, most likely occurring due to the absorption of oxygen from the -OH (allyl alcohol) plasma process exposure to air, and the 10-fold decrease in CF_2_. As a result, the introduction of the polar functional groups in the PTMem samples increased the wettability of a relatively inert and hydrophobic PTFE membrane, as evidenced by the ~2.5-fold increase in overall SE. However, the incorporation of oxygen into expanded PTFE grafts treated with plasma has been reported in the literature to result in different surface functionalities over time, which are difficult to predict [61,62].

From an in vitro cellular attachment standpoint, fluorescence analysis of the PTMem samples demonstrated significantly higher average cell population/mm^2^ in comparison to NTMem samples owing to the aforementioned physicochemical surface modifications. This is in agreement with other studies demonstrating limited cellular proliferation of human gingival and periodontal fibroblasts cultured upon unmodified PTFE membranes in vitro [63]. In the current study, a higher cell count was observed in the PTMem samples after 8 days, with significant differences observed as early as 1 day after cell seeding, relative to NTMem samples. This type of cell growth has also been reported in the literature, with similar elemental composition on the sample surface, such as oxygen and carboxyl groups, which enhanced endothelial cell growth [64] and cellular adherence [65], respectively. Briefly, the plasma–polymer surface phenomenon has been described as enabling greater hydrophilicity and provide reactive chemical functionalities that promote the binding of adhesion proteins, resulting in increased cellular attachment to polymeric surfaces with greater SE [66,67,68,69,70].

The current study highlights numerous beneficial surface physicochemical changes to PTFE membranes owing to the NTP process. However, it is also important to consider the limitations of this study. It has been indicated in the literature that an ideal bone regeneration membrane should be capable of maintaining its physical barrier function for 16–24 weeks to ensure the favorable biomechanical competence of regenerated bone [71]. In contrast, the current study evaluated the effect of NTP treatment on the PTFE membranes for up to 8 days. Additionally, the controlled environment of in vitro testing is vastly different from the environment of living tissues. While results from this study lend support to the modification of inert barrier membranes via NTP treatment for applications in GBR, they call for in vivo evaluation in preclinical trials. Furthermore, it is well known that plasma modification of polymer membranes is a surface phenomenon that yields chemical and structural changes to only a few nano- or micro-meters in depth, as reported in numerous studies [72,73,74,75]. Therefore, bulk properties (mechanical, thermal, and degradation profile) of the NTP treated samples have been reported to be similar to those of the control (non-treated) samples [72]. However, conclusive testing for these parameters falls outside of the scope of this current proof-of-concept study and warrants a follow-up evaluation via elaborate experimentation.

## 6. Conclusions

In contrast to unmodified hydrophobic PTFE (NTMem) membranes, our study demonstrated increased cellular adhesion and the proliferation of cells seeded on plasma-treated PTFE (PTMem) membranes. Improved physiochemical characteristics, such as the incorporation of functional groups and increased surface energies, were observed on PTMem samples. The results of the study support the potential applicability of NTP treatment to polymer biomaterials such as PTFE, which have a long history in clinical use as dental barrier membranes.

## Figures and Tables

**Table 1:** SE characterization of the two polymeric experimental groups used in this study.

Experimental Group	Parameter	Measured Value (Mean ± 95% Confidence Interval (CI)) (All Values in N/m)
NTMem	SE	15.0 ± 3.4
	Dispersive	15.0 ± 3.5
	Polar	0.11 ± 0.2
PTMem	SE	37.0 ± 4.8
	Dispersive	34.0 ± 6.3
	Polar	2.9 ± 2.0

**Table 2:** Atomic concentrations detected via XPS on the polymeric samples’ surface (in %). XPS did not detect hydrogen (H) or helium (He). A dash line “-” indicates that the element was not detected. A less-than symbol “<” indicates that accurate quantification could not be carried out due to weak signal intensity.

Group	Sample	C	O	F	Si	Cl
NTMem	1	33.7	0.4	65.8	0.1	<0.05
	2	31.0	0.2	68.9	-	<0.05
	3	30.9	-	69.0	-	<0.05
PTMem	1	73.0	19.7	7.1	0.1	<0.05
	2	66.3	17.0	16.7	-	<0.05
	3	75.1	21.2	3.4	0.3	<0.05

**Table 3:** Quantification of the carbon chemical state (in atom %) of the polymeric samples used in this study.

Group	Sample	C-C	C-O	C=O	O-C=O	CF	CF_2_	CF_3_
NTMem	1	2.6	0.4	0.3	0.0	0.5	28.0	1.9
	2	0.8	0.3	0.2	0.0	0.4	27.2	2.1
	3	0.3	0.2	0.2	0.0	0.5	27.9	1.9
PTMem	1	49.2	17.7	3.4	0.8	0.0	1.8	0.0
	2	41.1	15.7	2.9	0.7	0.0	6.0	0.0
	3	50.6	19.1	3.7	1.1	0.0	0.7	0.0

## Data Availability

The data that support the findings of this study are available from the corresponding author upon reasonable request.
